# Correlations Between Achilles Tendon Stiffness and Jumping Performance: A Comparative Study of Soccer and Basketball Athletes

**DOI:** 10.3390/jfmk10020112

**Published:** 2025-03-28

**Authors:** Daniel Schmidt, Lukas Verderber, Andresa M. C. Germano, Nico Nitzsche

**Affiliations:** 1 Motor Control, Cognition, and Neurophysiology, Chemnitz University of Technology, Thueringer Weg 11, 09126 Chemnitz, Germany; daniel.schmidt@hsw.tu-chemnitz.de (D.S.); lukas.verderber@hsw.tu-chemnitz.de (L.V.); andresa.germano@hsw.tu-chemnitz.de (A.M.C.G.); 2 Professorship of Sports Medicine and Exercise Therapy, Chemnitz University of Technology, Thueringer Weg 11, 09126 Chemnitz, Germany

**Keywords:** Achilles tendon stiffness, drop jump, basketball, soccer, correlation

## Abstract

**Background/Objectives**: Human tendon properties influence athletic performance, and it was shown that Achilles tendon (AT) stiffness correlates with an athlete’s jumping performance across sports. However, the findings on this relationship between basketball and soccer are different. Hence, this study examined the relationship between AT stiffness and jumping performance in male athletes. **Methods**: Sixty-six males (24.9 ± 4.7 years; twenty-two basketball players (22.0 ± 4.1 years), and forty-four soccer players (26.3 ± 4.4 years)) participated. Reactive jumping performance (reactive strength index (RSI), jump height (JH), and ground contact time (GCT)) were assessed using drop jumps (fall height: 30 cm), and AT stiffness (supine position) was measured using the MyotonPro. **Results**: Soccer players had a significantly higher AT stiffness (826.8 ± 90.5 N/m) than basketball players (754.1 ± 80.1 N/m, *p* = 0.002), but no differences were found in JH, RSI, or GCT (*p* > 0.05). JH and AT stiffness significantly correlated in basketball players (r = 0.448) but not in soccer players (r < 0.100). The multiple linear regression indicated that AT stiffness is significantly influenced by the sport type (soccer or basketball), while age, mass, and height remained non-significant. **Conclusions**: Despite higher AT stiffness in soccer players (which can be explained by different activity regimens), a moderate correlation between jumping performance and AT stiffness was evident only in basketball. Given the versatile demands of both sports, tendon characteristics appear to have an influence on jumping performance. For future studies, investigating tendon characteristics represents a valuable addition to training and therapy scheduling.

## 1. Introduction

Team sports are globally popular, with soccer and basketball being among the most practiced. Soccer has around 200 million players [1] and over 3.5 billion fans [2], while basketball is played by over 450 million people [3]. Injuries, especially to the lower extremities, are common in both sports [4,5]. Tendon injuries, including those to the Achilles tendon (AT), are frequently reported [6]. AT injuries may result from sudden strain on the plantar flexor muscle–tendon unit, such as landing after jumping [7].

Explosive power is vital in these sports. In basketball, vertical jumping is key to performance, with players performing 41–49 jumps per game depending on their position [8,9,10,11,12,13]. In soccer, power and jumping ability are also important [12,13,14]. However, the general requirements, as well as the load and stress protocol of soccer, differ from those of basketball [15]. In contrast to soccer, basketball requires primarily an anaerobic–lactacidic muscle metabolism, exhibits less functional splitting, and more jump events. There are also differences in terms of playing time or running distances [15]. Furthermore, the underground conditions in outdoor soccer (flexible and natural field) and in basketball (more or less rigid indoor underground) differ. Hence, there might be a varying profile of the dynamics of kicks, changes of direction, and other movement patterns depending on the underground properties (and/or different footwear). Other differences may also emerge from the different footwear employed in both sports. Although related to soccer only, an earlier investigation found that elite indoor players exhibited lower vertical jump and half-squat power compared to elite outdoor players [16].

Tendons once thought to adapt slowly [17], respond to exercise by increasing collagen synthesis [18]. The AT plays a critical role in explosive movements, transmitting force, or storing and recoiling energy [19,20], underscoring its importance in foot functionality, like plantar flexion [21]. Hence, tendons are often examined to determine athletic performance [22,23], especially during jumping, where limited time to produce force is present [24]. Importantly, tendons adapt to activities like jumping and landing due to internal structural changes [18,19,24].

To understand tendons in various scenarios, certain properties are commonly assessed, including strain, cross-sectional area, and Young’s modulus. Techniques like ultrasound and magnetic resonance imaging are used to measure tendon elongation or cross-sectional area [25,26,27,28]. Tendon stiffness, which describes a length change relative to the applied force, is another key property [18]. Myotonometry is one approach for assessing tendon stiffness, offering advantages such as low cost, high accuracy, good reliability, and a non-invasive operation [3,29,30].

Research suggests that higher AT stiffness is associated with reduced electromechanical delay in prepubertal children (10 boys and 10 girls) [31], with an increased torque in 16 male participants [32], and with an improved jump performance in 13 men [24]. AT mechanical properties, including stiffness, influence force transmission and the efficiency of the stretch–shortening cycle, which are critical for explosive movements like jumping. In 73 young male basketball players, small to moderate associations have been reported between drop jump performance and the gastrocnemius–Achilles tendon unit [3]. Similarly, in 15 adult male K-7 League soccer players, significant correlations have been observed between AT stiffness and vertical jump performance [33]. However, some studies did not assess a relationship between AT stiffness and jump performance (e.g., in first-league male (*n* = 12) basketball players [34] or in 21 soccer players [35]) or reported mixed results [36,37]. In this regard, Abdelsattar et al. [36] found negative correlations between AT stiffness and ground contact times in drop jumps but no association with squat jump or countermovement jump height in 19 physically active young males. Conversely, Konrad and Paternoster [37] found no correlation between AT stiffness and drop jump performance in 16 recreationally active males.

Despite the importance of AT properties for jumping ability, investigations on the relationships between AT stiffness and vertical jump performance comparing basketball versus soccer are not available. Furthermore, these relationships are generally different in other study populations. To our knowledge, no studies have compared soccer and basketball in this context, despite the relevance of AT stiffness for training planning. In this context, injury prevention and/or enhancing athletic performance constitute potential intersections based on such data. Given that basketball players rely heavily on vertical jumping while soccer players perform more multidirectional movements, differences in AT properties may lead to sport-specific adaptations. Therefore, this study aimed to quantify AT stiffness in players of both sports and to investigate its relationship with vertical jump performance. We hypothesized a different relationship between AT stiffness and jumping performance between the two sports.

## 2. Materials and Methods

### 2.1. Participants

Sixty-six healthy male athletes (22 basketball players and 44 soccer players) participated in this study (Table 1). The average training load per week was 4.0 ± 2.6 h for soccer players and 7.8 ± 2.0 h for basketball players. The average years of experience were 15.6 ± 8.1 years for soccer players and 7.2 ± 3.2 years for basketball players. All athletes were recruited from regional sports clubs in Saxony/Germany, and both sports groups were composed of high-level and intermediate-level players. For the basketball players, *n* = 10 were recruited from the first national league (high-level basketball players), and the remaining *n* = 12 from the youth first national league (intermediate-level basketball players). Among the soccer players, *n* = 19 were recruited from the first local soccer league in the state of Saxony (high-level soccer players), and *n* = 25 from the fourth local soccer league of the same state (intermediate-level soccer players). Some participants had experienced sport-related injuries in the past (such as muscle, tendon, or ligament injuries, bruises) but were free of injuries at data collection and at least 6 months prior to testing. Based on calculations using G-Power (version 3.1.9.7) incorporating data from related studies [3,24,25,32,33], a sample size of *n* = 20 per group (total *n* = 40) was required, excluding a possible dropout rate. Due to mainly logistical reasons, an elevated number of soccer players could be sampled. The G-Power calculations were performed with respect to correlations (bivariate normal model, α = 0.05, power range 0.7–0.8) and difference analyses (Anova: fixed effects, omnibus, one-way, two groups; power 0.8) (for details on data analyses, see Section 2.3).

Measurements were taken as a part of performance diagnostic tests, with all athletes providing informed written consent for participation in this study and for their data to be used for scientific purposes. This study was conducted in accordance with the Declaration of Helsinki (latest version) and the implemented methods were approved by the ethics committee of the Chemnitz University of Technology (V-267-17-HS-RSG-2404202018, date: 24 April 2020; V-197-17-DS-Wasser-16052017, date: 29 May 2017).

### 2.2. Protocol

After capturing the anthropometric data, participants went through an approx. 10 min warm-up process, including a bicycle ergometer (0.8 W/kg) and stretching of the lower extremities (e.g., quadriceps femoris, biceps femoris, gastrocnemius). The subsequent testing procedures were performed by two experienced experimenters, who were instructed specifically with regard to the corresponding measurement setup and performed several pilot tests prior to data collection on volunteers not enrolled in this study. This ensured that the performance of the tests implemented in this study was standardized and consistent.

**Figure 1:**
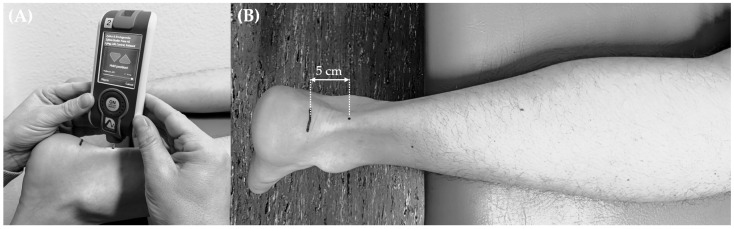
(**A**) Illustration of the measurements using the MyotonPRO device. (**B**) The Achilles tendon constituted the test location, 5 cm cranial from the calcaneal tuberosity.

The measurement of the Achilles tendon stiffness (N/m) was carried out using a MyotonPRO device (Myoton AS, Tallinn, Estonia) in the supine position (Figure 1). The measuring point was located 5 cm cranial from the tuberosity of the calcaneus. The ankle joint was positioned in a neutral zero position [38], and the muscles involved were not contracted [39]. Each measurement was taken three times per leg side by an experienced person. The average of all three measurements per leg side was used for data analysis. A force of 0.18 N (stimulus duration: 15 ms), with an angle of 90°, was applied to the tissue being tested during each measurement [3]. Finally, the calculated average per leg side was used for analyses.

**Figure 2:**
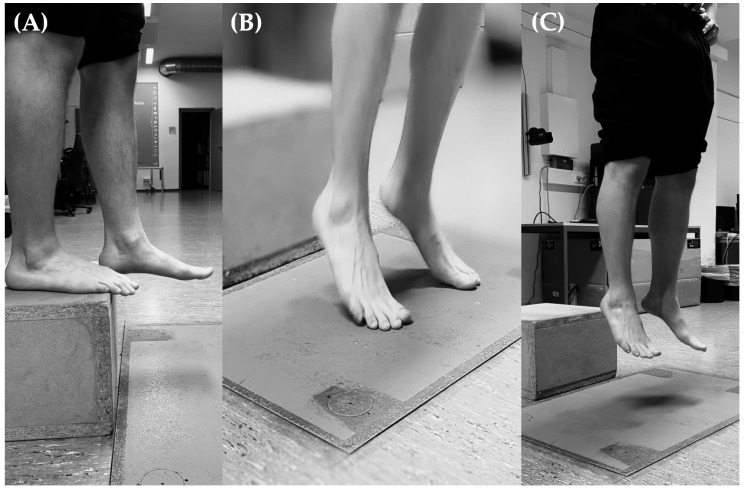
An exemplary illustration of a drop jump performed: (**A**) starting position at the edge of the box (fall height 30 cm), (**B**) toe contact with the ground (force plate), and (**C**) take-off/flight phase after initial ground contact.

Finally, all athletes were examined for their reactive strength and tissue resistance in the Achilles tendon area. This was performed after a jump-specific warm-up protocol: five two-legged, easy submaximal reactive jumps to have them become used to the protocol of the jumps. Subsequently, there were two test jumps, including feedback from the experimenter regarding the standardization requirements (see below). To measure the reactive strength, bipedal drop jumps (Figure 2, fall height: 30 cm) were performed on a force plate (force plate 86 × 60 cm, in-house construction based on four “Druckkraft Waegzelle V10S” (BOSCHE, sampling rate 1 kHz) and a DAQ-card (MCC USB-231)). A LabVIEW routine was used to calculate jump height based on the vertical force-time signal. By integrating acceleration over time, the velocity at takeoff was obtained. Jump height was then derived using a kinematic equation based on the velocity at take-off and the gravitational acceleration. Three drop jumps were performed bilaterally under standardized conditions. These include the starting position: standing upright, hands on hips, and stepping off without actively pushing. For the toe contact, landing on the forefoot with extended knees, a consistent landing position, and achieving the best shortest ground contact time were important. For the take-off/flight phase, aiming a maximum jump height, keeping the body upright, and maintaining a consistent push-off mechanics/posture were relevant. Approximately 60 s of rest was given between each jump [40]. The reactive strength index (RSI) was used as a measure of reactive strength, calculated based on jump height (cm) and ground contact time (GCT, in ms) [40]. The most effective drop jump with the highest RSI was used for further analysis. We did not randomize the order of the testing sequence (warm-up, stiffness measurements, and drop jump performance) to avoid detrimental effects on Achilles tendon stiffness.

### 2.3. Data Processing and Statistical Analyses

Data processing and analyses were carried out using R (The R Project for Statistical Computing). For the parameters stiffness, jump height, RSI, and GCT, intra-class correlations (ICCs) were calculated to assess relative reliability. According to Weir 2005 [41], model 3,1 was used because (a) ICCs were calculated based on the individual three measurements of each parameter (not their mean), and (b) no systematic error was usually present among these three trials.

In terms of the internal consistency of data collection performed by the two experimenters, there were overall no major differences between the individual three trials for the stiffness values of the dominant limb and drop jump performance for both sports (namely jump height, RSI, and ground contact time—note again that the most effective drop jump with the highest RSI was used for main analyses). Furthermore, the mean ± SD ICC coefficient (relative reliability) comparing the individual three trials for all parameters and both sport types was 0.841 ± 0.091 (range 0.615–0.963). These results confirm the consistency throughout data collection.

Differences between both sports groups (basketball vs. soccer) were analyzed using an independent t-test or the Mann–Whitney U-test, depending on data distribution. Stiffness differences comparing the right vs. left and the dominant vs. non-dominant limb (assessed by oral questioning) for each group were carried out using the dependent t-test or the Wilcoxon test, again depending on data distribution (alpha = 0.05 for all tests). Effect sizes were calculated based on Cohen’s D or Cliff’s Delta, depending on what test was used. Spearman correlations between jump parameters (jump height, RSI, GCT) and AT stiffness were calculated with alpha = 0.05.

Multiple linear regression analyses were performed based on the data including all participants (*n* = 66). Tendon stiffness was considered the dependent variable, whereas jump height, ground contact time (GCT), relative strength index (RSI), age, mass, height, and sport type (soccer or basketball) constituted the independent variables. Prior to calculating the regression model, various assumptions were verified. First, the dependent variable was scaled using a metric/ratio scale. Second, residuals were normally distributed. This was confirmed by means of histograms, Q-Q-plots, and the Shapiro–Wilk test (*p* = 0.608). Third, the residuals were of homoscedastic nature as confirmed visually and by using the Breusch–Pagan test (*p* = 0.235). Fourth, participant data were checked for possible outliers by means of Cook’s distances. No outliers were detected when the criterion of a critical Cook Distance of one was applied [42]. Fifth, a possible multi-colinearity between the independent variables was examined. Based on a Spearman correlation, there were significant (*p* < 0.05) interactions between RSI and jump height (r = 0.784), RSI and GCT (r = −0.720), and height and mass (r = 0.681). In addition, the variance inflation factor (VIF) was calculated to quantify the extent of multi-colinearity, whereas values greater than 10 were considered critical. This was true for jump height (VIF = 31.8), GCT (VIF = 18.3), and RSI (VIF = 51.9). Consequently, jump height, GCT, and RSI were removed prior to calculating the regression model. Hence, the final independent variables were age, mass, height, and sport type (basketball or soccer). A minimal dataset of this study is provided as Supplemental Material.

## 3. Results

### 3.1. Intra-Class Correlations (ICCs)

For the basketball players, all ICCs were significant and resulted in the following coefficients: 0.961 (stiffness dominant leg, explanation see Section 3.2), 0.894 (JH), 0.840 (RSI), and 0.726 (GCT). For the soccer players, all ICCs were also significant with the following coefficients: 0.925 (stiffness dominant leg), 0.800 (JH), 0.801 (RSI), and 0.769 (GCT).

### 3.2. Analyses of Differences Between and Within the Two Sport Types

Achilles tendon stiffness did not differ between the right and left or the dominant and non-dominant limb of both sports groups. Hence, tendon stiffness of the dominant limb was used for the subsequent analyses.

**Figure 3:**
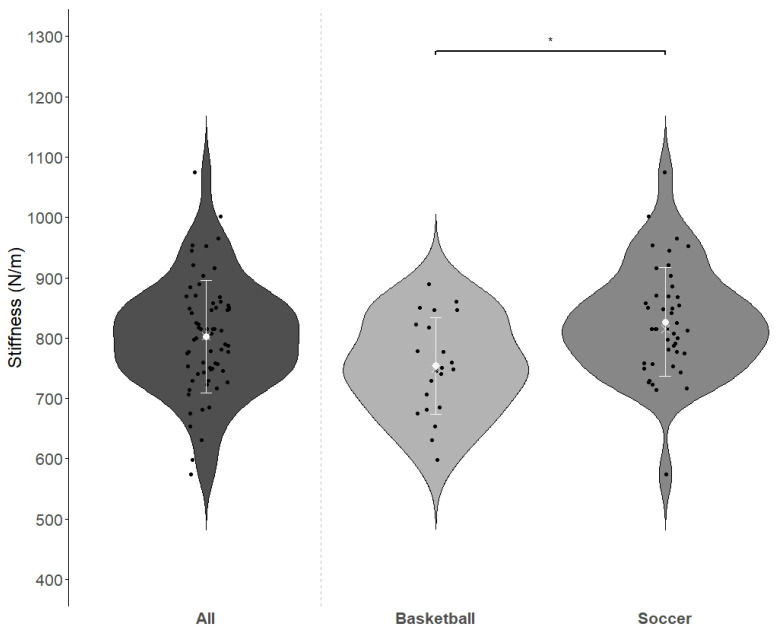
Violin plots of the Achilles tendon stiffness (N/m) for both groups (Basketball (*n* = 22), Soccer (*n* = 44), and the entire sample (All, *n* = 66)). Black dots: individual data points, white dot: mean, white cross: median, white error bars: standard deviation. Significant differences: * *p* = 0.002, Cohen’s D = 0.833.

Comparing jump-related parameters (jump height (JH), ground contact time (GCT), and relative strength index (RSI) as well as stiffness between both groups, soccer players exhibited significantly higher stiffness values compared to basketball players (*p* = 0.002, Cohen’s D = 0.833, see Table 2/Figure 3). The remaining parameters revealed no significant differences.

### 3.3. Analyses of Correlations

For soccer players, no correlations were observed between tendon stiffness of the dominant limb and jump-related parameters (Table 3). For the sake of completeness, we observed the same findings when considering the non-dominant, right, and left limbs. In addition to this aspect, there were significant correlations between GCT and jump height (r = −0.321), RSI and jump height (r = 0.768), and RSI and GCT (r = −0.816) for soccer players.

**Figure 4:**
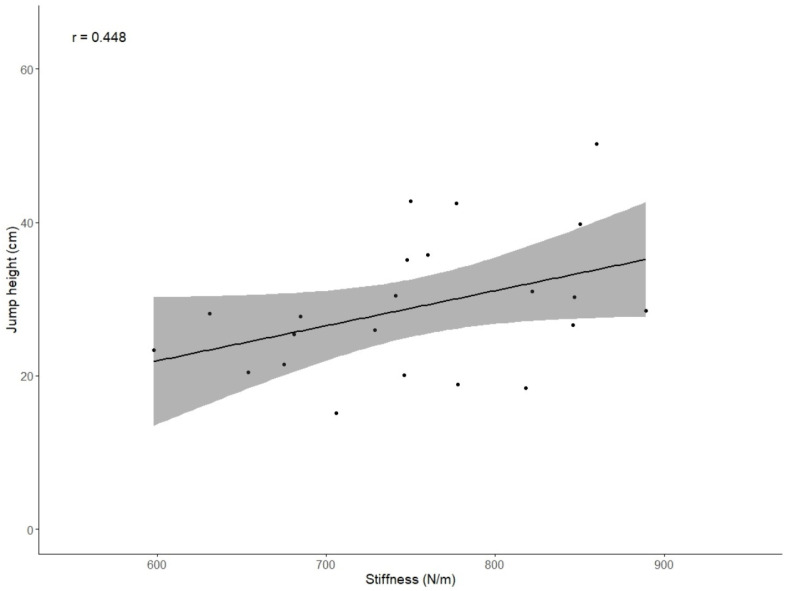
Spearman correlation plot between jump height (cm) and stiffness (N/m) for basketball players (r = 0.448, *p* = 0.038). The gray-shaded area represents the 95% confidence interval of the regression line.

For basketball players (Table 3/Figure 4), a significant correlation was confirmed between the tendon stiffness of the dominant limb and jump height (r = 0.448). As an addendum, it should be mentioned that correlations between jump height and the non-dominant, right and left limbs, were also significant (r = 0.680, r = 0.609, *p* = 0.485, respectively). Furthermore, there were significant correlations between RSI and jump height (r = 0.838) and between RSI and GCT (r = −0.624).

### 3.4. Multiple Linear Regression Analyses

The multiple linear regression model revealed a significant F-statistic (*p* = 0.007). The multiple R-squared (R^2^) value was 0.203, and the adjusted R-squared value was 0.151. The sport type displayed a significant *p*-value (*p* = 0.005), whilst the other variables remained non-significant (age: *p* = 0.869, mass: *p* = 0.250, and height: *p* = 0.854).

## 4. Discussion

### 4.1. Introductory Considerations Regarding AT Stiffness

Before discussing the results, we classified the AT stiffness data measured with the MyotonPRO. For basketball players, we found values (754 ± 80 N/m) similar to previous studies reporting 700–800 N/m for young players [3] and 727 ± 112 N/m for Polish league players [34].

For soccer players, we found 827 ± 91 N/m, which is lower than the elite players reported in Cristi-Sánchez et al. (1031–1075 N/m) [43], but comparable to amateur players reported in Seon et al. (800–1100 N/m) [33]. Other studies on active males [21,37] also show values around 808 N/m and 848 N/m, aligning with our results, despite variations induced by age, sportive activity and level, test sites, sample size, or the type of the (Myoton) device used.

### 4.2. ICCs

For the stiffness measures of the dominant limb, large ICC (model 3,1) coefficients of 0.961 (basketball) and 0.925 (soccer) were found, reflecting high relative reliability [44,45]. This is in line with other investigations [29,30]. We also calculated ICCs for pairwise comparisons of the three individual stiffness measurements (1 vs. 2, 1 vs. 3, 2 vs. 3) and found similarly high coefficients. For the other parameters (jump height, RSI, GCT), ICC coefficients were lower but still reflected a good overall relative reliability [46].

Acknowledging that a high magnitude of an ICC may simply stem from a high between-subject variability [41], we have calculated the corresponding coefficients of variation (COVs). We found high ICCs (e.g., 0.961) and corresponding lower COVs (e.g., 0.106) but also smaller ICCs (e.g., 0.840) and higher corresponding COVs (e.g., 0.386). This indicates that our ICC magnitudes indeed confirm an overall high/good relative reliability.

### 4.3. Side Differences

The lack of side-related stiffness differences is consistent with a previous study [21]. Furthermore, Cristi-Sánchez et al. [43] and Gervasi et al. [3] also found no differences within the limbs of soccer and basketball players, respectively.

### 4.4. Differences Between Soccer and Basketball Players

Our study showed a higher tendon stiffness in soccer players. This finding was supported by the multiple linear regression model showing that the sport type significantly influenced AT stiffness. The fact that tendon stiffness varies with different sports and/or activity levels was also found when comparing sprinters versus endurance runners [25] and soccer players versus controls [43].

It appears that the soccer-specific activity protocol (and not the basketball-specific protocol) triggers specific stimuli that increase AT stiffness. Although some of the basic training regimens and requirements of both sport types appear relatively similar (e.g., upper and lower extremity power, speed endurance, agility skills, or strength), different stress and strain profiles of soccer versus basketball activities are present. In competitions, soccer players complete more sprints (150 vs. 100), a longer game duration (50–60 vs. 40 min), a longer net playing time (60 vs. 50%), and greater running distances (8–14 vs. 4–5 km) compared to basketball players [15]. In addition to the features already provided in the introduction, there are also differences in the percentages of actual distances spent walking or sprinting during a game. Soccer players walk about 8% further than basketball players, and the sprint distance is approx. 5 times less than basketball players (calculations based on data provided in Ferrauti et al. 2020 [15]). It is likely that these and other load- and strain-specific differences persist in the training routines of both sports and explain the results of our study.

The increased stiffness in soccer players may be explained by the following mechanisms: physiologically, a minimum level of load intensity (strain amplitude or frequency) is necessary to trigger such changes [18,25]. This adaptation is seen in both the tendon’s material properties (e.g., stiffness) and its morphology (e.g., cross-sectional area) [18,26]. Changes in material properties primarily result from alterations in collagen fibril structure, increased cross-linking between collagen molecules, and higher collagen synthesis [18].

As mentioned earlier, both sports groups were heterogeneous in terms of experience and playing level. High-level basketball players (8.3 ± 2.3 years of experience) were more proficient than intermediate-level soccer players (18.1 ± 5.4 years of experience). Intermediate sub-groups also differed in proficiency and experience (basketball: 6.3 ± 3.6 years, soccer: 13.7 ± 9.4 years). High-level groups showed no parameter differences, whereas intermediate-level soccer players had significantly greater stiffness, higher JH, and longer GCT than basketball players. For JH, this aligns statistically with the previous findings (descriptively for most of the other parameters, see Table 2).

Incorporating the sportive level into the regression analysis showed that both sport type and skill level influence tendon stiffness. These findings should, however, be interpreted with caution. Due to the differing sportive experience and skill level (e.g., high-level basketball players vs. high-level soccer players), direct comparisons may be difficult. An ultra-sound-based study found that long-term training and/or experience may modulate tendon adaptations, such as stiffness [47]. Furthermore, the number of participants per group considerably dropped after splitting. Hence, we did not include this aspect in our main analyses and this sub-section is for additional information only.

### 4.5. Correlation Analyses

The correlation between AT stiffness and jump height in basketball players (suggesting that jump height may be influenced by AT stiffness) was also found by Gervasi et al. [3]. Furthermore, they found no interaction with ground contact time but a significant correlation between RSI and GAT stiffness [3]. The contrasting findings may be due to the lower age of their participants (12–18 years), whereas tendon stiffness increased with increasing age [3]. Interestingly, Gervasi et al. [3] found that the stiffness of the AT decreases with an increase in distance from the plantar aspect of the heel.

The correlations in their study apply only to distal tendon locations (8–12 cm from the heel), not proximal ones (16 and 20 cm). Our test location aligns with the distal areas studied by Gervasi et al. [3], making both studies comparable. Ando et al. [48] similarly found a correlation between medial gastrocnemius stiffness and jump height (and RSI, unlike our findings), but not ground contact time (GCT). The lack of correlation between GCT and AT stiffness may be due to the given instructions; that is, both Gervasi et al. [3] and our study had participants focus on jumping as high as possible. This instruction leads to a highly individual force development, likely preventing a systematic pattern in GCT correlations [3].

For soccer players, there were no significant correlations between AT stiffness and jump variables (height, GCT, RSI). This supports our hypothesis of different relationships between AT stiffness and jump parameters when comparing soccer and basketball. The absent relationship in soccer contrasts with the results from Seon et al. [33], who found a significant correlation between AT stiffness and jump height in soccer players, although they did not specify where exactly stiffness was measured (note again that AT stiffness varies along the tendon [3]). Additionally, Seon et al. [33] used counter-movement jumps, which rely more on knee extensors, while drop jumps primarily involve the triceps surae [36], possibly explaining the differing results.

Studies on non-soccer/basketball athletes also showed correlations between tendon stiffness and jump parameters. In active males, AT stiffness negatively correlated with ground contact time in drop jumps [36], contrasting with our findings. Similar to our soccer player results, Abdelsattar et al. [36] found no correlation between AT stiffness and jump height, although they used squat and counter-movement jumps and assessed AT stiffness via ultrasound during isometric contractions. These methodological differences likely explain the varying results.

Bojsen-Møller et al. [32] found a correlation between vastus lateralis tendon stiffness (ultrasound-based) and jump height in cyclists and volleyball players, aligning with our basketball results. Similarly, Burgess et al. [24] found a correlation between medial gastrocnemius stiffness and jump height in men without specifying athletic background. In contrast, Konrad and Paternoster [37] found no correlation between myotonometric AT stiffness and drop jump performance in active males (similar to our soccer findings), possibly due to their short ground contact times (<250 ms, range: 133–225 ms) following extensive conditioning. This shows that varying study results must be interpreted with the inclusion of certain co-parameters.

In light of that, Konrad and Paternoster [37] suggest that longer GCTs (150–350 ms [36] and 148–290 ms [48]), linked to less conditioning/practice, may explain correlations between jump height and tendon stiffness found [36,48]. Our study showed GCTs of 151–366 ms for soccer players and 147–308 ms for basketball players, with no statistical difference (*p* = 0.073). While GCTs were comparable to those in previous studies, we found correlations only in basketball players. This suggests that varying GCTs or drop-jump-specific skills, as proposed by Konrad and Paternoster [37], do not explain the differing results. The lack of correlation in soccer players might be due to their lower and more variable weekly training load compared to basketball players. Hence, despite the higher soccer-specific load and stress profile, the lack of correlations may partially be explained by this fact. Alternatively, one could also follow a possible explanation mentioned in [36]: the lower limb muscle–tendon unit does not seem to be the major source for jumping movements. From this perspective, the relationship between the AT stiffness and jump height does not appear to be substantial. However, we are currently unable to provide further potential explanations for this finding, particularly the discrepancy between soccer and basketball.

In terms of the correlation analyses mentioned above, we also considered the two sub-groups for each sport type (following the principle described in the last paragraph of Section 4.4). Each soccer-specific and basketball-specific sub-group did not show any significant correlations between AT stiffness vs. jump height, ground contact time, and RSI (all *p* < 0.244, all r < |0.406|). This contrasts the findings described above (correlation between jump height and AT stiffness for all 44 basketball players) but is in line with [36]. The reasons for this discrepancy may, in addition to the above-mentioned paragraphs, similarly be due to the heterogeneous sportive level/type/sportive experience and/or the smaller sample size due to sub-group splitting. Hence, this observation must be interpreted with caution and represents additional information only.

To conclude, this study aimed to quantify AT stiffness and drop-jump-related parameters (jump height, ground contact time, and RSI) in soccer and basketball players and to investigate their relationships and sport-type-related differences. We generally hypothesized varying relationships. We found that soccer players showed significantly greater AT stiffness compared to basketball players. A significant correlation between jump height and AT stiffness was found in basketball players but not in soccer players, supporting our hypothesis and the literature. Multiple regression analysis revealed that AT stiffness is significantly influenced by the type of sport. The reasons for the differing relationships between AT stiffness and jump parameters in soccer and basketball, as well as tendon and stiffness differences between both sports, remain mostly speculative based on the data we collected.

### 4.6. Limitations

To better control the influences of muscle tension, the inclusion of electromyography for the lower limb muscles would be able to control the resting state during MyotonPro measurements [49]. Similarly, the measurement may be influenced by subcutaneous adipose tissue [49]. Since the plantar flexor muscles, in particular, play an important role in executing drop jumps, assessing muscle stiffness would also contribute.

In addition, our sample revealed that basketball players were, on average, slightly older than soccer players, which may have an impact on correlations with jumping performance [50]. In order to control the hormonal influences depending on the time of measurement, the measurements were carried out in the afternoon. It is also important to note that the MyotonPro provides a measurement of tissue resistance and is used as a surrogate for tendon stiffness [51,52]. We also did not have information regarding a detailed training routine for our participants (e.g., jumping or running periods/quantity). In addition, in our main analyses, we did not further differentiate between the sportive level within each of the two sports groups (e.g., professional versus semi-professional). Although we did this to avoid lowering the corresponding sample size, possible effects of the level of proficiency might have been masked. Differences in such parameters have the potential to affect study outcomes and should be addressed in future investigations.

Another aspect is related to the fact that our participants in both sports groups had several different playing positions. For soccer, these were goalkeeper (*n* = 3), defense (*n* = 11), midfield (*n* = 15), and forward (*n* = 15). For basketball, these were small forward (*n* = 6), point guard (*n* = 6), power forward (*n* = 4), shooting guard (*n* = 4), and center (*n* = 2). It is likely that these differ in terms of physical and psychological demands, resulting in varying biomechanical properties between players. Interestingly, a related study found that in elite soccer players, AT stiffness did not vary when comparing different playing positions [43]. The same publication concluded that “Although players in other positions may display different physical demands, the loads and the training dosage that they were subjected to during years of professional training would be the same”. Considering that in basketball, functional position splitting is clearly less prominent compared to soccer [15], we assume that the different playing positions among our participants may not affect the interpretation of our results.

Finally, our sample sizes were biased (22 basketball players versus 44 soccer players), which might have affected outcomes. However, we wanted to include as many participants as possible in order to obtain more robust and representative values. We also performed a bootstrap approach (resampling approach, 1000 iterations/repetitions (with replacements), interpretation based on mean differences between groups and 95% confidence intervals) comparing both sports groups for the parameters jump height and AT stiffness. For jump height (*n*_Soccer_ = 22), the mean difference was 3.35 cm, and the confidence interval included negative values (−1 to 7.4 cm). This indicates that the (rather small) difference is likely not robust, which is in alignment with the absence of significant differences compared to when including *n* = 44 participants. For the AT stiffness (*n*_Soccer_ = 22), the mean difference was 71.5 N/m, and the confidence intervals were positive (22.5 to 118.5 N/m). This suggests that soccer players likely exhibit a higher stiffness than basketball players, which is in line with the results obtained when implementing *n* = 44 participants (soccer). Taking this together, we found that a random sample of 22 soccer players (or a random sample of 44 basketball players) yielded similar findings. Hence, the biased sample size does not appear to be problematic.

## Figures and Tables

**Table 1:** Mean ± SD anthropometric data of the participants.

	Age (years)	Height (m)	Mass (kg)	BMI (kg/m^2^)
Basketball players (*n* = 22)	22.0 ± 4.1 *	1.91 ± 0.1 *	84.7 ± 13.7 *	23.1 ± 1.9
Soccer players (*n* = 44)	26.3 ± 4.4 *	1.79 ± 0.1 *	75.5 ± 7.5 *	23.5 ± 1.8
*p*-value	* ˂0.001	* ˂0.001	* 0.007	0.417
Effect size	^δ^ 0.512	^δ^ −0.649	^D^ −0.925	^D^ 0.219
All (*n* = 66)	24.9 ± 4.7	1.83 ± 0.1	78.6 ± 10.8	23.4 ± 1.9

Significant differences are marked with “*”, effect sizes were calculated based on Cliff’s Delta (^δ^) or Cohen’s D (^D^).

**Table 2:** Mean ± SD of the examined parameters jump height (JH), ground contact time (GCT), relative strength index (RSI), and stiffness of the dominant leg.

	JH (cm)	GCT (ms)	RSI	Stiffness (N/m)
Basketball players (*n* = 22)	29.0 ± 9.0	211.9 ± 41.8	1.4 ± 0.5	754 ± 80 *
Soccer players (*n* = 44)	32.4 ± 5.7	235.0 ± 46.5	1.4 ± 0.4	827 ± 91 *
*p*-value	0.114	0.074	0.935	* 0.002
Effect size	^D^ 0.494	^δ^ 0.273	^D^ 0.023	^D^ 0.833
All (*n* = 66)	31.3 ± 7.1	227.3 ± 46.0	1.4 ± 0.4	803 ± 93

Significant differences are marked with “*”, effect sizes were calculated based on Cliff’s Delta (^δ^) or Cohen’s D (^D^).

**Table 3:** Spearman correlation coefficients (r) (and *p*-values) between Achilles tendon stiffness (Stiffness, N/m), jump height (cm), ground contact time (GCT, ms), and relative strength index (RSI) for soccer and basketball players.

	Spearman r (*p*-Value)	
Comparison	Soccer	Basketball
Stiffness vs. Jump height	−0.147 (0.341)	0.448 (0.037) *
Stiffness vs. GCT	0.060 (0.699)	0.319 (0.148)
Stiffness vs. RSI	−0.104 (0.500)	0.148 (0.511)
Jump height vs. GCT	−0.321 (0.003) *	−0.133 (0.554)
Jump height vs. RSI	0.768 (0.500)	0.838 (<0.001) *
GCT vs. RSI	−0.816 (<0.001) *	−0.624 (0.002) *

* significant correlations.

## Data Availability

The raw data supporting the conclusions of this article are attached.

## Supplementary Materials

The following supporting information can be downloaded at: https://www.mdpi.com/article/10.3390/jfmk10020112/s1.
